# Association of Arterial Stiffness (Brachial–Ankle Pulse Wave Velocity) with Angiographic Outcomes After Drug-Eluting Stent Implantation: A Retrospective Lesion-Level Cohort Study

**DOI:** 10.3390/jcm15176613

**Published:** 2026-08-27

**Authors:** Hangyeol Kim, Hyun Sung Joh, Woo-Hyun Lim, Hack-Lyoung Kim, Sang-Hyun Kim, Jae-Bin Seo

**Affiliations:** 1 Department of Internal Medicine, Seoul National University Hospital, Seoul National University College of Medicine, Seoul 03080, Republic of Korea; penofblack@gmail.com; 2 Department of Cardiology, Seoul National University Boramae Medical Center, Seoul 07061, Republic of Korea; wingx4@naver.com (H.S.J.); woosion@gmail.com (W.-H.L.); khl2876@gmail.com (H.-L.K.); shkimmd@snu.ac.kr (S.-H.K.)

**Keywords:** arterial stiffness, brachial–ankle pulse wave velocity, acute gain, late lumen loss, quantitative coronary angiography, drug-eluting stent

## Abstract

**Background/Objectives:** Elevated brachial–ankle pulse wave velocity (baPWV) predicts adverse outcomes after percutaneous coronary intervention, but its relation to the quantitative angiographic outcomes of drug-eluting stent (DES) implantation is unknown. We examined baPWV against acute gain and late lumen loss. **Methods:** In a retrospective single-center cohort, 242 lesions in 141 patients with paired quantitative coronary angiography (QCA) were analyzed. baPWV was modeled per 1 SD with patient-level clustering (linear mixed model for acute gain; cluster-robust ordinary least squares for late lumen loss), with a Bonferroni-corrected α = 0.025 for the co-primary outcomes. Generalizability was assessed against registry patients without QCA. **Results:** An unadjusted inverse association with acute gain (β = −0.093 mm per 1 SD; *p* = 0.013) was fully attenuated after adjustment (β = −0.019; 95% CI −0.106 to +0.068; *p* = 0.67). baPWV was not associated with late lumen loss (adjusted β = −0.021; 95% CI −0.091 to +0.050; *p* = 0.57). **Conclusions:** Pre-procedural baPWV showed no independent association with acute gain or late lumen loss after DES implantation, consistent with a marker of systemic cardiovascular risk rather than independent local angiographic information. These data do not support using baPWV to guide procedural decisions.

## 1. Introduction

Arterial stiffness, evaluated by brachial–ankle pulse wave velocity (baPWV), is a well-established independent predictor of cardiovascular morbidity and mortality [1,2]. In patients with acute coronary syndrome, elevated baPWV predicts major adverse cardiac events independently of traditional risk factors [3], and in stable patients referred for coronary computed tomography angiography, it adds incremental prognostic value beyond the anatomical burden of atherosclerosis [4]. Mechanistically, increased arterial stiffness raises pulse pressure and pulsatile arterial load, hemodynamic changes linked to adverse coronary outcomes, including restenosis [5].

Among patients undergoing percutaneous coronary intervention (PCI) with drug-eluting stent (DES) implantation, baPWV has been consistently linked to long-term clinical outcomes. In a single-center Korean cohort of 372 PCI-treated patients, a high baPWV (≥1672 cm/s) predicted cardiac death [6]. In a registry of 3930 PCI-treated patients, a high baPWV (≥1891 cm/s) was independently associated with greater 4-year risks of net adverse clinical events (adjusted HR 1.44; 95% CI 1.12–1.85), major adverse cardiac and cerebrovascular events (HR 1.40; 95% CI 1.07–1.83), and major bleeding (HR 1.94; 95% CI 1.15–3.25) [7]; one-month changes in blood-pressure-adjusted baPWV further stratify risk [8].

Beyond clinical events, three small studies have linked arterial stiffness to in-stent restenosis (ISR), a binary angiographic endpoint whose distinct patterns carry different long-term prognoses [9]. In 126 patients receiving bare-metal stents, the aortic stiffness index (ASI) was the strongest independent predictor of ISR (OR 6.8; 95% CI 2.6–13.5) [10]. In a mixed revascularization cohort of 160 patients (PCI and coronary artery bypass grafting), pulse wave velocity was higher in those who developed ISR [11]. More recently, in the DES era, a higher pulse-pressure index independently predicted ISR among 644 patients [12].

All prior studies linking arterial stiffness to stent outcomes have relied on either binary in-stent restenosis or clinical event endpoints. Additionally, because baPWV is tightly correlated with age and cardiometabolic burden, no study has established whether arterial stiffness carries information about the angiographic response to stenting, which is independent of these shared risk factors. Whether baPWV is a modifiable mechanistic contributor to the stented-vessel response or merely a downstream marker of global cardiovascular risk remains unknown, this distinction is decision-relevant: baPWV is inexpensive, non-invasive, and already widely measured in routine cardiovascular practice. If pre-procedural baPWV values were independently associated with acute gain or late lumen loss, it could inform lesion preparation, device sizing, post-dilation strategy, and the intensity of angiographic surveillance; conversely, demonstrating that they add no independent angiographic information would argue against their use for such procedural decisions and refocus their role on systemic risk stratification. No study has examined this question using the quantitative coronary angiography (QCA) metrics of acute gain and late lumen loss, which offer finer mechanistic resolution than threshold-based ISR. Resolving it—whichever direction the association takes—has both mechanistic and practical value.

We therefore examined whether pre-procedural baPWV is independently associated with the quantitative angiographic response to DES implantation, as captured by acute gain and late lumen loss.

## 2. Materials and Methods

### 2.1. Study Design and Pre-Specified Hypotheses

This was a retrospective, lesion-level cohort study designed to test two co-primary hypotheses: that higher pre-procedural baPWV is independently associated with lower acute gain (Hypothesis 1; β < 0) and with greater late lumen loss (Hypothesis 2; β > 0) after DES implantation. Critically, we asked whether any such association persists after adjustment for the clinical and angiographic factors with which baPWV co-varies—that is, whether baPWV adds independent information about the stent response beyond conventional risk markers. The hypotheses, the covariate set, and the analysis plan were fixed before the analysis was undertaken, and each co-primary test used a Bonferroni-corrected two-sided α = 0.025 (Section 2.6).

### 2.2. Setting and Ethics

The study was conducted at the Cardiovascular Center, Seoul National University Boramae Medical Center, Seoul, Republic of Korea, and was approved by the Institutional Review Board of the same institution (approval number 06-2012-93). Individual informed consent was waived under the retrospective de-identification protocol. The study complied with the Declaration of Helsinki and is reported in accordance with the STROBE guidance for observational studies [13]. The lesion-level quantitative coronary angiography dataset analyzed here does not overlap with the cohorts reported in the group’s prior brachial–ankle pulse wave velocity outcome studies [6,7,8], which examined clinical events rather than angiographic restenosis endpoints.

### 2.3. Participants, QCA Cohort, and the Nature of the Missing Data

**Figure 1:**
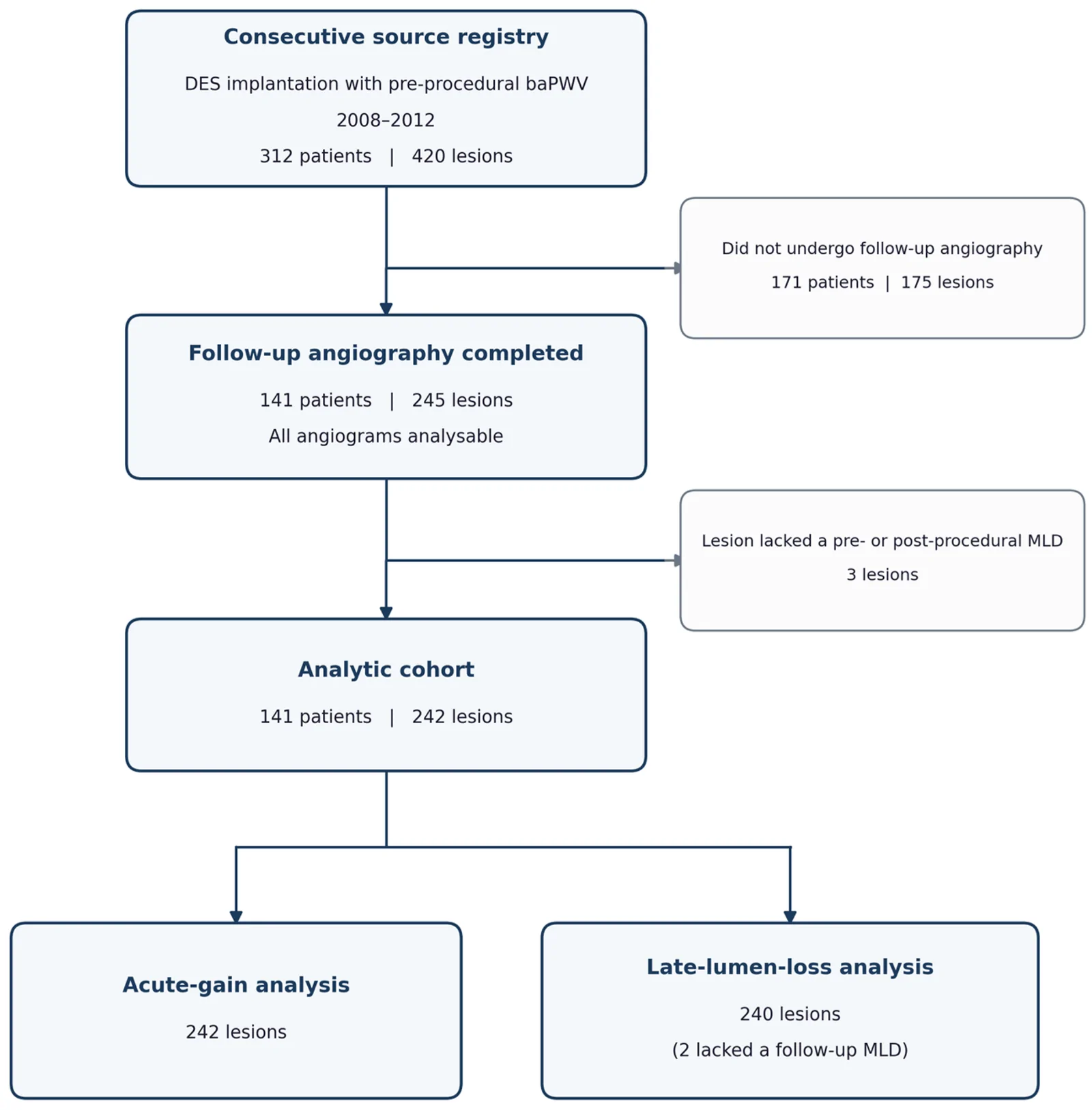
STROBE participant flow: baPWV registry (420 lesions/312 patients) → QCA cohort (242 lesions/141 patients; acute gain 242, late lumen loss 240).

We studied consecutive patients who underwent drug-eluting stent (DES) implantation with pre-procedural brachial–ankle pulse wave velocity (baPWV) at the Cardiovascular Center, Seoul National University Boramae Medical Center, between August 2008 and March 2012 and in whom quantitative coronary angiography (QCA) was performed. The study population comprised the 141 patients (242 lesions) with complete paired pre-procedural, post-procedural, and follow-up QCA, which define the lesion-level angiographic endpoints (median angiographic follow-up, 280 days [IQR 273–306]). This cohort was drawn from a consecutive source registry of 312 patients (420 lesions) with baPWV. The 171 patients (175 lesions) who did not undergo follow-up angiography are compared with the study cohort for generalizability (Table S1). Both chronic and acute coronary syndromes were eligible (acute coronary syndrome, 72% of the study cohort), and there were no additional lesion-based exclusion criteria beyond the performance of QCA. Follow-up angiography at approximately nine months was scheduled for every patient under the institutional surveillance protocol rather than prompted by symptoms, and 141 of the 312 registry patients (45%) attended. Non-attendance was at the patient’s discretion, and no patient was excluded by the investigators. No angiogram was unavailable and none was unsuitable for quantitative analysis. Participant flow is summarized in Figure 1.

### 2.4. Exposure

Pre-procedural baPWV was evaluated with an automated oscillometric device (VP-1000; Collin Co., Ltd., Komaki, Japan) and expressed as the mean of the right and left measurements in cm/s [14,15]. Measurements were obtained during the index admission as part of the routine vascular work-up, either on the day before the procedure (day −1) or on the day of the procedure (day 0). Patients rested in a supine position for at least five minutes in a quiet, temperature-controlled room before cuffs were applied to both arms and both ankles, in accordance with the standard measurement protocol for this device [14]. Mean blood pressure at the time of measurement was 130 ± 22/74 ± 11 mmHg and mean heart rate was 67 ± 13 bpm. Patients were not excluded on the basis of peripheral arterial disease, and the ankle–brachial index was below 0.9 in 15 patients (10.6%); arrhythmia status was not recorded in the source registry. Measurement conditions and the corresponding sensitivity analyses are detailed in Table S5. For the primary lesion-level analyses, baPWV was Z-standardized using the lesion-level distribution of the analytic cohort (mean 1643 ± 354 cm/s, 1 SD = 354 cm/s), so that coefficients represented the change per 1-SD increment. The corresponding patient-level distribution was 1637 ± 357 cm/s.

### 2.5. Outcomes and Covariates

Acute gain was defined as immediate post-PCI minus pre-PCI minimum lumen diameter (MLD), and late lumen loss was defined as immediate post-PCI minus follow-up MLD [16]. Two of the 242 lesions lacked a follow-up minimum lumen diameter and were excluded from the late-lumen-loss analysis (*n* = 240). QCA was performed by experienced observers blinded to baPWV values.

Pre-specified covariates were age, sex, diabetes mellitus, hypertension, current smoking, estimated glomerular filtration rate, reference-vessel diameter (acute-gain model), pre-PCI MLD (late-lumen-loss model and an acute-gain ANCOVA sensitivity analysis), and follow-up duration (late-lumen-loss model). The covariate set was fixed in advance, and selection of covariates by univariable significance was prohibited [17].

Left ventricular ejection fraction (LVEF) was measured by transthoracic echocardiography during the index admission. The source registry did not record a discrete diagnosis of heart failure, so left ventricular function is described by LVEF alone; its ascertainment, distribution, and the corresponding post hoc analyses are reported in the Supplementary Materials (Text S2, Tables S1, S3 and S4).

### 2.6. Statistical Analysis

Acute gain was analyzed with a linear mixed model (random intercept per patient, restricted maximum likelihood). Late lumen loss was analyzed with cluster-robust ordinary least squares because its within-patient correlation was low. baPWV was modeled continuously (per 1 SD), and each co-primary test used a Bonferroni-corrected threshold (α = 0.025, two-sided). The unadjusted association was summarized with Pearson and Spearman correlation (Fisher z-transformed 95% confidence intervals), and the functional form of the exposure-outcome relationship was examined with a locally weighted scatterplot smoother (LOESS) displayed against the linear fit.

Several pre-specified analyses examined robustness. The baPWV estimate was cross-validated across the linear mixed model, cluster-robust OLS, and generalized estimating equations and, because late lumen loss was right-skewed (skewness 1.81), confirmed with distribution-robust methods (signed-square-root transform, Huber regression, and rank-based regression). Because the study population was defined by the performance of QCA, we did not project estimates to the source registry but instead compared patients with and without QCA to assess generalizability (Table S1). The two lesions lacking a follow-up minimum lumen diameter were handled by complete-case analysis (late lumen loss, *n* = 240). All pre-specified covariates were complete in the study cohort. Reference-vessel diameter (defined as the post-procedural reference diameter) and every model covariate were available for all 242 lesions. Because baPWV is a patient-level exposure with limited independent information, the available procedural and device covariates were added parsimoniously (singly, with the stent-sizing variables as a single block) rather than in one saturated model.

Analyses were performed in R 4.5.2 (lme4, clubSandwich, and geepack) and Python 3.11.15 (statsmodels), with large-sample (normal-approximation) inference. We did not pre-specify a formal equivalence margin. Accordingly, a non-significant co-primary result whose 95% confidence interval excluded effects of clinically relevant magnitude was interpreted as indicating the absence of a clinically important association rather than as formal proof of equivalence or as an inconclusive (underpowered) result. No a priori sample-size calculation was performed. All eligible patients with pre-procedural baPWV and paired QCA were included, and statistical precision was summarized by the width of the confidence intervals.

### 2.7. Use of Generative Artificial Intelligence

During the preparation of this manuscript the authors used a generative artificial-intelligence tool (Claude.ai; Anthropic, San Francisco, CA, USA) solely for language editing and formatting assistance. The tool was not used to design the study, to collect or curate the data, to perform or verify any statistical analysis, or to interpret the results; all analyses were specified and executed by the authors in R and Python as described above. All artificial-intelligence-assisted text was reviewed and edited by the authors, who take full responsibility for the content of the publication.

## 3. Results

### 3.1. Cohort and Baseline Characteristics

The analytic cohort comprised 242 lesions in 141 patients (mean 1.72 lesions per patient). The mean age was 66.0 ± 10.3 years. Overall, 66.7% were male, 40.4% had diabetes, 66.0% had hypertension, and 25.5% were current smokers (Table 1). Acute gain was approximately normally distributed (mean 1.48 ± 0.55 mm), whereas late lumen loss was right-skewed (skewness 1.81) and is summarized as the median (0.05 mm, IQR −0.14 to 0.28). Median angiographic follow-up was 280 days (IQR 273 to 306). Higher baPWV was associated with older age, more frequent diabetes and hypertension, and lower eGFR.

### 3.2. Unadjusted Associations and Functional Form

**Figure 2:**
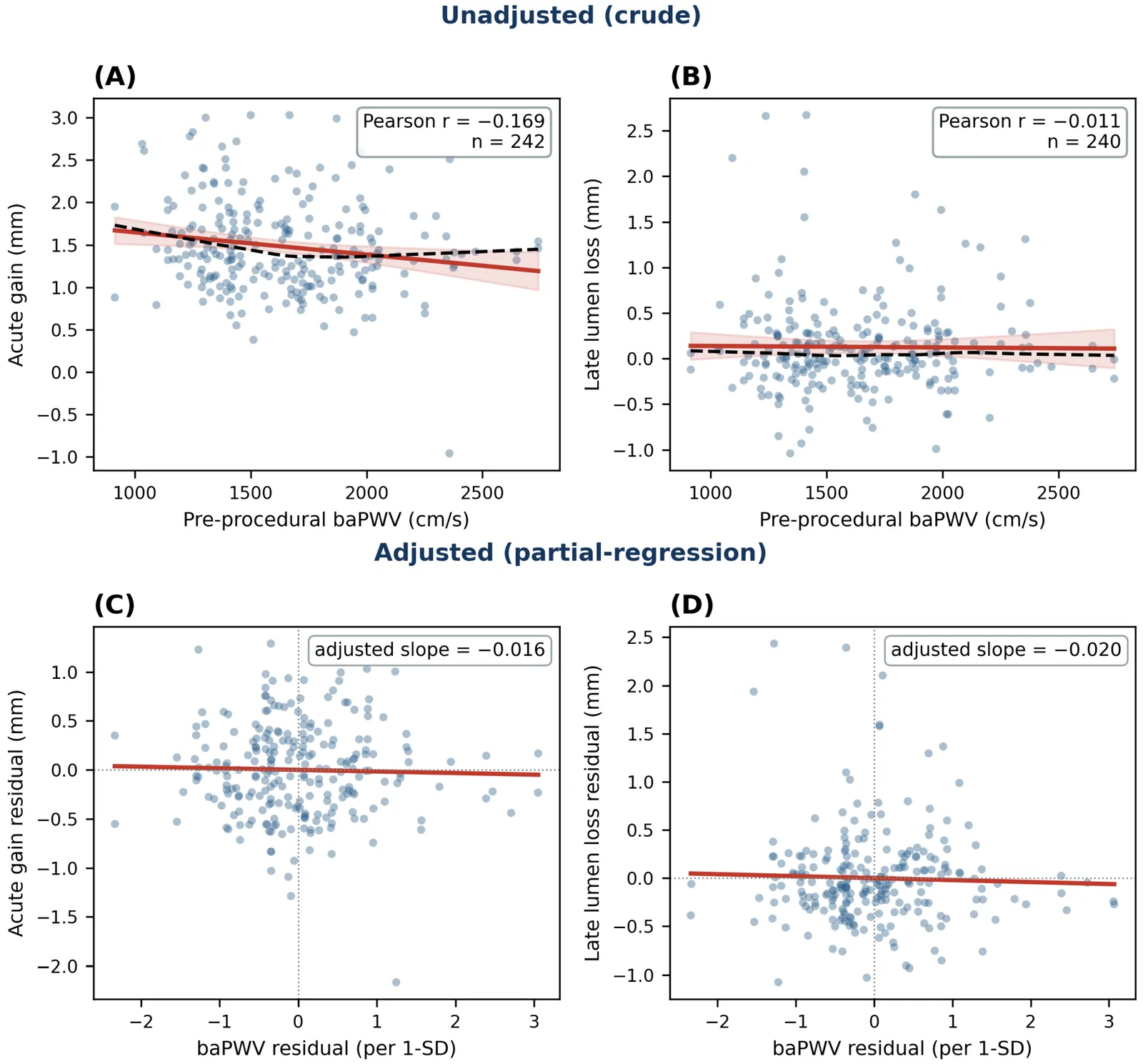
Relationship of pre-procedural baPWV with acute gain and late lumen loss, before and after adjustment. The top row shows unadjusted scatterplots for (**A**) acute gain and (**B**) late lumen loss, with the linear fit and its 95% confidence band in red and a LOESS as a dashed line. The bottom row shows partial-regression (added-variable) plots of the adjusted relationship for (**C**) acute gain and (**D**) late lumen loss, in which outcome residuals are plotted against baPWV residuals after regression on the model covariates, so that the slope of the red line equals the covariate-adjusted coefficient. The crude inverse association between baPWV and acute gain in (**A**) is abolished after adjustment in (**C**), whereas late lumen loss is flat in both (**B**,**D**). Each point is a lesion. Formal inference is from the clustered regression models (Table 3).

In unadjusted analysis, baPWV was weakly but significantly inversely correlated with acute gain (Pearson r = −0.169, 95% CI −0.289 to −0.044, *p* = 0.008) and essentially uncorrelated with late lumen loss (Pearson r = −0.011, 95% CI −0.14 to +0.12, *p* = 0.86), and the Spearman coefficients were concordant. These are unadjusted correlations, and the corresponding cluster-robust regression estimates appear in Table 2. On the scatterplots (Figure 2), acute gain declined approximately linearly with increasing baPWV whereas late lumen loss remained flat, and the LOESS tracked the linear fit closely, with no indication of meaningful non-linearity.

### 3.3. Attenuation After Adjustment: Confounding by Shared Common Causes

**Figure 3:**
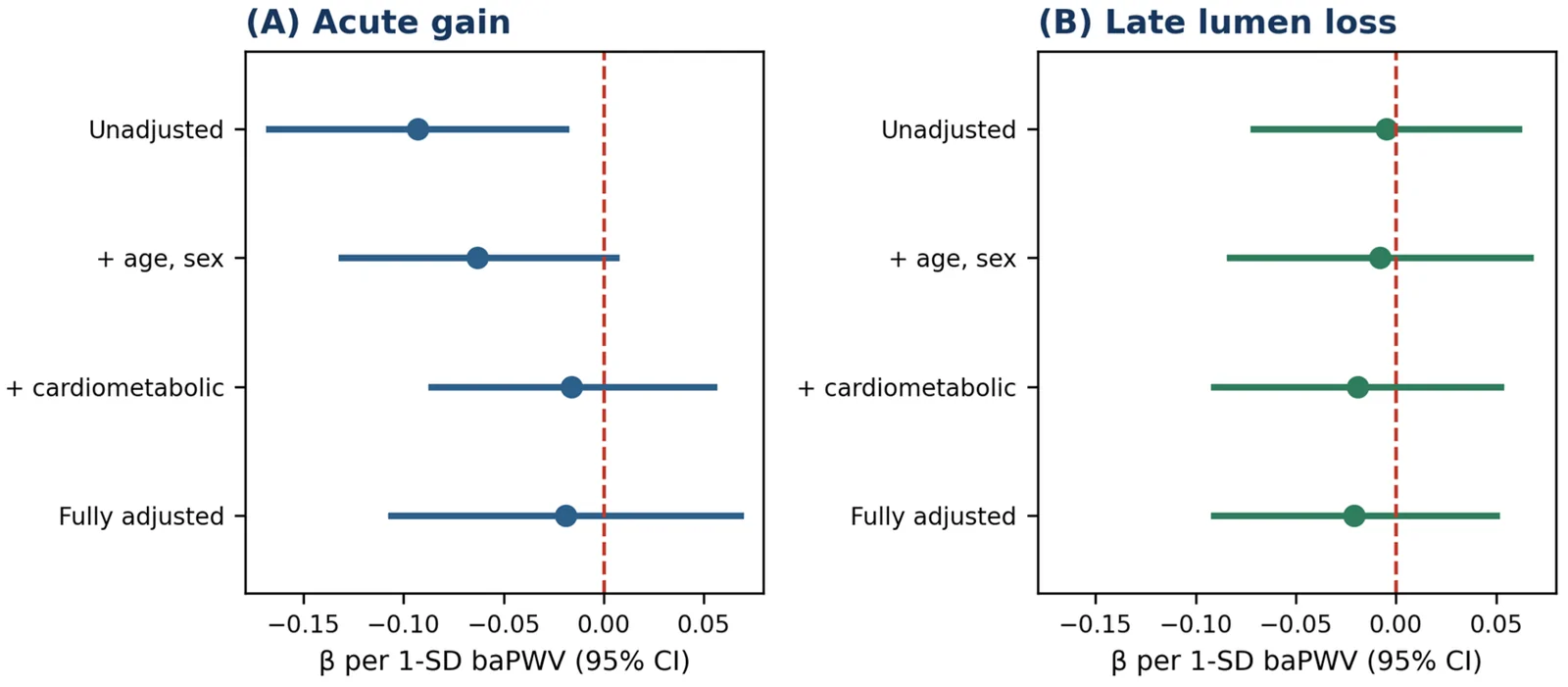
Attenuation of the baPWV coefficient with progressive adjustment. Points are the baPWV regression coefficient (β per 1 SD) with 95% confidence intervals at each stage of adjustment for (**A**) acute gain and (**B**) late lumen loss, and the dashed red line marks the null. For acute gain, the significant crude association moves to the null once age and cardiometabolic factors are added, consistent with confounding by shared common causes. Late lumen loss shows no association at any stage. Values correspond to Table 2.

The acute-gain association depended entirely on adjustment. In unadjusted analysis, higher baPWV was significantly associated with lower acute gain (β = −0.093 mm per 1 SD, *p* = 0.013). This association was progressively and completely attenuated to the null as age and the other cardiometabolic factors (diabetes, hypertension, current smoking, and eGFR) were added, and reference-vessel diameter contributed little further change (fully adjusted β = −0.019, *p* = 0.67; Table 2 and Figure 3). Late lumen loss showed no association at any level of adjustment. These adjusted relationships are shown as partial-regression plots in Figure 2C,D. baPWV is itself correlated with age (r ≈ 0.34), diabetes (r ≈ 0.26), and hypertension (r ≈ 0.28), each an established determinant of the angiographic result. The crude acute-gain signal is therefore best interpreted as a process of confounding by these shared common causes rather than as an effect of arterial stiffness on the stented segment.

### 3.4. Co-Primary Results

Consistent with this attenuation, neither co-primary test reached significance at α = 0.025 in the fully adjusted models (Table 3). baPWV was not associated with acute gain (β = −0.019 mm per 1 SD, 95% CI −0.106 to +0.068, *p* = 0.67; intraclass correlation 0.30) or with late lumen loss (β = −0.021 mm per 1 SD, 95% CI −0.091 to +0.050, *p* = 0.57). The result was unchanged in the pre-PCI-MLD ANCOVA sensitivity analysis for acute gain (β = −0.015, *p* = 0.46), in the distribution-robust analyses of late lumen loss (Table S2), and across the linear mixed model, cluster-robust OLS, and GEE estimators (agreement within 0.003 mm per 1 SD).

The null also persisted after adjustment for available procedural and device covariates, whether stent length, number, and diameter were added as a single block (acute gain β = −0.014, late lumen loss β = −0.013) or each procedural and lesion-size factor was added singly (all β between −0.011 and −0.036, all *p* ≥ 0.16). These sensitivity models are reported in Table S3.

### 3.5. Generalizability

Patients in the QCA cohort had modestly lower arterial stiffness than registry patients without QCA (baPWV 1637 vs. 1728 cm/s, standardized mean difference −0.23), together with somewhat less frequent hypertension and acute coronary syndrome but otherwise similar characteristics (Table S1), so the findings apply primarily to patients selected for QCA.

## 4. Discussion

In this retrospective cohort of 242 lesions (141 patients) undergoing DES implantation at a single Korean center, pre-procedural baPWV was not independently associated with either acute gain or late lumen loss after adjustment for established clinical and angiographic confounders. The null was consistent across all pre-specified sensitivity analyses, including the distribution-robust analyses. The 95% confidence intervals excluded associations larger than about 0.11 mm per 1 SD for acute gain and 0.09 mm per 1 SD for late lumen loss. For late lumen loss, this bound lies below the roughly 0.1 mm difference associated with meaningful changes in restenosis risk [18], so any true association is likely smaller than a clinically relevant threshold. Two caveats temper this interpretation. No formal equivalence margin was pre-specified, and the acute-gain bound lacks a comparably established clinical anchor, so it should be read more cautiously than the late-lumen-loss bound. However, because baPWV is a purely patient-level exposure (within-patient SD = 0), the effective information for its coefficient is governed by the number of patients (*n* = 141) rather than lesions.

### 4.1. Interpretation and Mechanisms

Several mechanisms may explain the absence of an independent association despite biological plausibility. First, baPWV co-varies strongly with age and cardiometabolic risk factors. The unadjusted acute-gain association largely reflected these shared common causes, so baPWV carried little information about the angiographic outcomes beyond that of established risk factors. Second, acute gain is standardized by the operator through device sizing to the reference vessel and post-dilation to a target residual stenosis, which attenuates biologically driven variation. Moreover, calcified, stiff lesions that resist expansion often prompt more aggressive lesion preparation, a procedural compensation that can offset a true effect. Third, baPWV is blood-pressure dependent and was measured once, so nondifferential measurement error biases the coefficient toward the null (regression dilution). Finally, baPWV is a systemic organism-level index, whereas acute gain and late lumen loss are local, lesion-specific phenomena, so only a modest correlation would be expected a priori.

### 4.2. Comparison with the Prior Literature

Our findings sit between two largely separate bodies of evidence. The more robust body links baPWV to clinical events after PCI, namely net adverse clinical events, major adverse cardiac and cerebrovascular events, and major bleeding in a 3930-patient registry [7], consistent with earlier cohorts [6,8]. A smaller and more heterogeneous body of literature links arterial stiffness to binary in-stent restenosis (ISR), reporting the aortic stiffness index in bare-metal-stent patients [10], pulse wave velocity in a mixed revascularization cohort [11], and a pulse-pressure index in DES patients [12]. To our knowledge, no prior study has examined baPWV against the continuous quantitative-angiographic metrics of acute gain and late lumen loss, which isolate the local stent response with finer resolution than threshold-based restenosis. These prior studies differ from the present analysis along two axes, endpoint type (binary threshold versus continuous late lumen loss) and stiffness construct (conduit stiffness versus hemodynamic load versus clinical events), as summarized in Table 4.

Several considerations reconcile our null with this prior work. Drug elution strongly suppresses neointimal proliferation, compressing late lumen loss (mean 0.13 mm here) and the variance through which a host factor such as stiffness could express itself, an effect likely greater with contemporary devices than in the bare-metal era. The prior reports also used different and not necessarily interchangeable surrogates (aortic stiffness index, pulse wave velocity, pulse-pressure index) and were generally small, so their estimates should be interpreted with some caution. In addition, binary restenosis is a threshold event that depends jointly on neointimal growth and baseline vessel caliber, and it collapses angiographically distinct patterns with different long-term prognoses into a single category, whereas late lumen loss more directly reflects the neointimal and recoil process itself, the histopathology of which has been characterized in detail [19]. Although the two are related at the population level [18], that relationship is modulated by reference-vessel diameter. Arterial stiffness associations with restenosis may therefore reflect this caliber dependence rather than an effect on neointimal biology, consistent with the attenuation of the crude association between baPWV and acute gain after adjustment for age and cardiometabolic risk factors in our data (Table 2).

Taken together, these observations are most consistent with baPWV acting as a systemic marker of cumulative vascular aging rather than a factor carrying independent information about the stented-segment response. Its prognostic value after PCI [6,7,8] is more plausibly mediated by system-level pathways, such as atherosclerotic burden, microvascular dysfunction, adverse left-ventricular remodeling, and bleeding propensity [20,21], than by focal stent mechanics, although these mediators were not examined here. The present study thus helps localize the predictive power of baPWV to the patient and the system rather than the lesion.

### 4.3. Clinical Implications

For daily practice, these findings place baPWV at the level of the patient rather than the lesion. Pre-procedural baPWV should not be used to anticipate a suboptimal acute result or to justify more aggressive lesion preparation, device upsizing, or routine post-dilation. The fully adjusted upper bound for acute gain, approximately 0.11 mm per 1 SD, is smaller than the smallest increment separating available stent diameters (0.25 mm) and so could not alter a sizing decision. Decisions about lesion preparation and stent optimization should continue to rest on lesion-level information such as vessel size, plaque burden, calcium morphology, and the achieved angiographic and intravascular-imaging result, rather than on a systemic stiffness index.

Additionally, nor should baPWV be used to select patients for angiographic surveillance, because it was unrelated to late lumen loss at every level of adjustment. That point carries weight, because routine follow-up angiography itself provokes ‘oculostenotic’ reintervention without demonstrated benefit [22] and is not recommended in asymptomatic patients after PCI [23].

These findings do not diminish the value of baPWV, but they relocate it. A high pre-procedural value remains a legitimate prompt to intensify systemic secondary prevention, including blood-pressure control, lipid lowering, glycemic management, and smoking cessation. Its result relates to the patient’s cardiometabolic risk-factor management rather than the catheterization laboratory decision tree, and the null lesion-level finding and robust patient-level prognostic literature are complementary rather than contradictory.

### 4.4. Strengths

This study has several methodological strengths. We analyzed lesion-level data with patient-level clustering using mixed models, with concordant estimates across modeling approaches. We explicitly addressed the principal threats to validity. The right-skewed late-lumen-loss distribution was handled with distribution-robust methods, the marker-versus-mechanism question with a transparent unadjusted-to-adjusted estimation cascade, and the generalizability of the QCA-defined cohort with a transparent comparison against patients without QCA that defined the population to which the findings apply. The use of continuous QCA endpoints rather than threshold-based restenosis increased mechanistic resolution. Because the primary finding is null throughout, the numerous sensitivity analyses guard against a fragile null rather than inflating the risk of a false-positive claim, supporting interpretation of the result as a reasonably informative null rather than a negative study.

### 4.5. Limitations

This study has several limitations. First, the retrospective, single-center design precludes causal inference and limits generalizability, because the referral and procedural patterns of a single center may not extend to other settings. Second, the study population was defined by the performance of QCA, which was available for 242 of 420 registry lesions. Because patients undergoing QCA had somewhat lower arterial stiffness, hypertension, and acute coronary syndrome, the findings generalize primarily to such patients. Late lumen loss was missing for only two lesions within the cohort and was handled by complete-case analysis. Third, baPWV is a patient-level exposure measured once before the procedure. Because it is blood-pressure dependent, residual confounding by unmeasured blood-pressure variation may persist despite hypertension adjustment. Fourth, we lacked data on lesion calcification and post-dilation, which could modify stent expansion and the neointimal response independently of arterial stiffness. However, the available procedural and device variables (stent length, stent number, stent diameter, lesion length, pre-procedural diameter stenosis, and DES type) were examined in sensitivity analyses without changing the findings (Table S3), and antiplatelet type was uniform (aspirin 100%, clopidogrel 99%; Table 1) and so could not confound.

The selection imposed by protocol-driven angiographic surveillance is more consequential for external validity. Routine follow-up angiography at approximately nine months belongs to the previous era in which this cohort was assembled (2008–2012) and is no longer standard practice. Current guidelines do not recommend it in asymptomatic patients [23], in part because it prompts reintervention for lesions of uncertain functional significance [22]. Patients who complete such a protocol are therefore selected. They must survive, remain well enough to return, and consent to a further invasive procedure. Nonetheless, the same design feature is the principal strength of this study, because uniform, symptom-independent angiographic endpoints in every lesion are what an unbiased continuous analysis of late lumen loss requires. These findings are therefore internally valid for the mechanistic question posed, whereas their quantitative extrapolation to contemporary, imaging-guided practice requires confirmation.

The lack of intravascular imaging also warrants consideration. Acute gain and late lumen loss were derived entirely from quantitative coronary angiography, which renders the lumen silhouette but not the vessel wall. Plaque composition, calcium burden, stent expansion, malapposition, and edge dissection are therefore invisible in our data, although each influences the acute result and subsequent neointimal growth. Minimal stent area is among the strongest predictors of angiographic patency at nine months [24], and an optical coherence tomography calcium score predicts stent under-expansion [25]. Intravascular ultrasound or optical coherence tomography would have permitted two analyses that angiography cannot support. One is whether baPWV tracks the coronary substrate itself rather than the net lumen result that the operator titrates toward a target. The other is whether apparent under-expansion instead reflects a genuinely small vessel. This distinction also helps explain why a systemic marker may fail to track lesion-level angiographic outcomes, since any influence of arterial stiffness on the substrate is most plausibly expressed in wall-level features for which the operator compensates during the procedure, leaving little residual signal in the lumen. Given the randomized and meta-analytic support for intravascular imaging in complex PCI [26,27], future studies of host-level determinants of the stent response should be imaging-based.

The study period warrants specific comment. Patients were treated between 2008 and 2012, and device utilization was recorded per patient rather than per lesion. Consequently, lesions were classified based on the stent generations implanted in each patient: 170 of 242 lesions (70%) involved only second-generation drug-eluting stents (DES), 31 (13%) involved only first-generation devices, 36 (15%) involved both generations, and 5 (2%) could not be assigned to a specific generation. Contemporary clinical practice has since evolved, featuring advanced stent designs, the routine use of intravascular imaging and physiological lesion assessment, and optimized antiplatelet and lipid-lowering therapies, which collectively yield superior outcomes. Thus, the null findings observed in this cohort are unlikely to display a positive association with modern stents. However, the absolute angiographic values reported herein should not be directly extrapolated to current practice.

## 5. Conclusions

In this single-center cohort, pre-procedural baPWV was not independently associated with acute gain or late lumen loss after adjustment for clinical and angiographic confounders. The apparent unadjusted association with acute gain was fully explained by age and cardiometabolic risk factors, with reference-vessel diameter adding little further change. These findings provide no evidence that baPWV carries independent information about the local angiographic response to stenting beyond established clinical and angiographic risk factors, consistent with its role as a marker of systemic cardiovascular risk. They therefore do not support using baPWV to individualize procedural decisions such as lesion preparation, device sizing, post-dilation, or the intensity of angiographic surveillance. Its well-documented prognostic value after PCI is more plausibly mediated through systemic atherosclerotic burden and hemodynamic load than through focal stent mechanics.

These conclusions derive from a retrospective, single-center cohort restricted to patients who completed protocol-scheduled follow-up angiography and should be interpreted cautiously. Confirmation requires adequately powered, multicenter studies with complete angiographic follow-up. These studies should incorporate intravascular imaging, which characterizes the vessel wall and the achieved stent result at a level of detail that quantitative angiography cannot reach.

## Figures and Tables

**Table 1:** Characteristics of the analytic cohort (242 lesions in 141 patients).

Characteristic	Value
Patient-level (*N* = 141)	Patient-level (*N* = 141)
Age, years	66.0 ± 10.3
Male sex	94 (66.7%)
Diabetes mellitus	57 (40.4%)
Hypertension	93 (66.0%)
Current smoker	36 (25.5%)
eGFR, mL/min/1.73 m^2^	78.1 ± 23.9
LVEF, % (*n* = 136)	62.9 ± 11.1
LVEF ≥ 50%	121 (89.0%)
LVEF < 50%	15 (11.0%)
LVEF ≤ 40%	9 (6.6%)
Aspirin	141 (100%)
Clopidogrel	140 (99.3%)
baPWV, cm/s (patient-level)	1637 ± 357
Lesion-level (*N* = 242)	Lesion-level (*N* = 242)
baPWV, cm/s (lesion-level)	1643 ± 354
Pre-PCI MLD, mm	0.86 ± 0.49
Post-PCI MLD, mm	2.34 ± 0.40
Reference-vessel diameter, mm	2.72 ± 0.49
Acute gain, mm	1.48 ± 0.55 (mean)
Late lumen loss, mm (*n* = 240)	0.05 [−0.14, 0.28] (median); 0.13 (mean)
Follow-up duration, days (*n* = 240)	280 [273, 306] (median)

Values are mean ± SD, *n* (%), or median [IQR]. The patient-level baPWV SD (357) and the lesion-level baPWV SD (354, used for per-1-SD standardization) differ because of multiple lesions per patient. eGFR, estimated glomerular filtration rate; MLD, minimum lumen diameter; baPWV, brachial–ankle pulse wave velocity. LVEF was available in 136 of 141 patients (96.5%), and the LVEF percentages use 136 as the denominator; heart failure was not recorded as a discrete diagnosis (Text S2). LVEF, left ventricular ejection fraction.

**Table 2:** Unadjusted-to-adjusted estimation cascade (per 1 SD baPWV).

Model	Acute Gain β (95% CI; *p*)	Late Lumen Loss β (95% CI; *p*)
Unadjusted	−0.093 (−0.167 to −0.019; 0.013)	−0.005 (−0.071 to +0.061; 0.88)
+age, sex	−0.063 (−0.131 to +0.006; 0.075)	−0.008 (−0.083 to +0.067; 0.83)
+cardiometabolic risk factors	−0.016 (−0.086 to +0.055; 0.66)	−0.019 (−0.091 to +0.052; 0.60)
Fully adjusted	−0.019 (−0.106 to +0.068; 0.67)	−0.021 (−0.091 to +0.050; 0.57)

Models are cumulative. ‘+age, sex’ adds age and sex to the unadjusted model. ‘+cardiometabolic risk factors’ additionally adds diabetes, hypertension, current smoking, and eGFR. ‘Fully adjusted’ additionally adds reference-vessel diameter (and, for late lumen loss, pre-PCI MLD and follow-up duration). eGFR, estimated glomerular filtration rate; MLD, minimum lumen diameter.

**Table 3:** Association of baPWV (per 1 SD) with acute gain and late lumen loss.

Analysis	β (mm/SD)	95% CI	*p*
Acute gain, crude	−0.093	−0.167 to −0.019	0.013
Acute gain, ANCOVA sensitivity (pre-MLD)	−0.015	−0.053 to +0.024	0.46
Acute gain, primary (LMM, fully adjusted)	−0.019	−0.106 to +0.068	0.67
Late lumen loss, primary (cluster-robust OLS)	−0.021	−0.091 to +0.050	0.57

Bonferroni-corrected threshold α = 0.025. ANCOVA, analysis of covariance; LMM, linear mixed model; MLD, minimum lumen diameter; OLS, ordinary least squares.

**Table 4:** Interpretive comparison of prior arterial stiffness/restenosis studies and the present study.

Study	Stiffness Measure (Construct)	Outcome	Era/Device	*n*	Reported Finding	Interpretation Alongside the Present Null
Mahfouz [10]	Aortic stiffness index (conduit)	Binary ISR	BMS	126	OR 6.8 (2.6–13.5)	Small, imprecise; BMS-era neointimal variance; effect likely real but inflated and era/surrogate-specific
Prskalo [11]	Pulse wave velocity (conduit)	Binary ISR	Mixed PCI + CABG	160	PWV higher in ISR	Heterogeneous population; group comparison; hypothesis-generating
Huang [12]	Pulse-pressure index (load)	Binary ISR	DES	644	Independent predictor	DES era but load construct and threshold endpoint; vessel-caliber pathway plausible
baPWV-events [6,7,8]	baPWV (conduit)	MACE, bleeding, death	DES	≤3930	Independent predictor	Robust, but systemic clinical endpoint, supporting a marker, not a local-mechanism, role
Present study	baPWV (conduit)	Continuous QCA (acute gain, late lumen loss)	DES	141 (242 lesions)	Null (adjusted)	Continuous endpoint isolates neointima; confounding/selection-robust, no independent local effect

BMS, bare-metal stent; CABG, coronary artery bypass grafting; DES, drug-eluting stent; ISR, in-stent restenosis; MACE, major adverse cardiac events; PWV, pulse wave velocity.

## Data Availability

The de-identified data underlying the findings are not publicly available because they contain potentially identifying and sensitive patient clinical information and are subject to ethical and legal restrictions imposed by the Institutional Review Board (IRB) of Seoul National University Boramae Medical Center. A de-identified minimal data set is available to researchers who meet the criteria for access to confidential data, upon reasonable request and with appropriate ethical approval, from the IRB/Data Access Committee of Seoul National University Boramae Medical Center (contact: +82-2-870-1851). Requests should be directed to this institutional point of contact rather than to the individual authors. The analysis code (R 4.5.2 and Python 3.11.15) supporting the reported results is available from the corresponding author upon reasonable request.

## Supplementary Materials

The following supporting information can be downloaded at: https://www.mdpi.com/article/10.3390/jcm15176613/s1, Text S1: Supplementary methods; Text S2. Left Ventricular Function and Heart Failure; Table S1: Generalizability: comparison of patients with versus without QCA; Table S2: Distribution-robust analyses of late lumen loss (baPWV per 1-SD; complete cases, *n* = 240); Table S3: Procedural, device, and left-ventricular-function covariate sensitivity analyses (baPWV per 1-SD; each covariate added to the fully adjusted model); Table S4: Exploratory analysis of effect modification by left ventricular ejection fraction (baPWV per 1-SD); Table S5: Sensitivity analyses for peripheral arterial disease and blood pressure at measurement (baPWV per 1-SD).
