# Supplementary material for: Association of Arterial Stiffness (Brachial–Ankle Pulse Wave Velocity) with Angiographic Outcomes After Drug-Eluting Stent Implantation: A Retrospective Lesion-Level Cohort Study
Source: J Clin Med. 2026 Aug 27;15(17):6613. doi: 10.3390/jcm15176613 (PMC13566099; doi:10.3390/jcm15176613)
Supplement: Supplementary file 1 [file jcm-15-06613-s001.zip › jcm-4488537-supplementary.pdf]

Supplementary Materials

Association of Arterial Stiffness (Brachial–Ankle Pulse Wave Velocity) with Angiographic Outcomes (Acute Gain and Late Lumen Loss) after Drug-Eluting Stent Implantation: A Retrospective Lesion-Level Cohort Study

Hangyeol Kim, Hyun Sung Joh, Woo-Hyun Lim, Hack-Lyoung Kim, Sang-Hyun Kim, Jae-Bin Seo

Contents.

Text S1 summarizes the estimation framework and the generalizability comparison of patients with and without QCA, and Text S2 describes left ventricular function and the ascertainment of heart failure.

Table S1 reports the generalizability comparison, Table S2 the distribution-robust late-lumen-loss analyses, Table S3 the procedural, device, and left-ventricular-function covariate sensitivity analyses, and Table S4 an exploratory analysis of effect modification by left ventricular ejection fraction. Table S5 reports the corresponding sensitivity analyses for ankle-brachial index and mean blood pressure at the time of baPWV measurement.

Text S1. Supplementary Methods

Linear mixed models were fitted by restricted maximum likelihood; cluster-robust (sandwich) standard errors used patient as the clustering unit; and the selection model was a logistic regression. The baPWV estimate was additionally checked with an exchangeable-working-correlation generalized estimating equation.

Because the study population was defined by the performance of QCA, estimates were not projected to the source registry. The two lesions within the cohort lacking a follow-up minimum lumen diameter were handled by complete-case analysis (late lumen loss, n = 240). Generalizability was assessed by comparing patients with and without QCA (Table S1).

Text S2. Left Ventricular Function and Heart Failure

Left ventricular ejection fraction (LVEF) was measured by transthoracic echocardiography during the index admission. It was available in 136 of the 141 patients with QCA (96.5%) and in 158 of the 171 registry patients without QCA (92.4%). In the analytic cohort the mean LVEF was 62.9 ± 11.1% (median 65%, interquartile range 56–71%): 121 patients (89.0%) had an LVEF of 50% or more, 6 (4.4%) between 41% and 49%, and 9 (6.6%) of 40% or less, so 15 patients (11.0%) had an LVEF below 50%. Percentages use the 136 patients with an available measurement as the denominator.

The source registry does not contain a discrete diagnosis of heart failure or a heart-failure hospitalization endpoint. It was assembled between 2008 and 2012 to relate brachial–ankle pulse wave velocity to ischaemic and mortality outcomes, and the adjudicated clinical events are cardiac death, non-cardiac death, acute myocardial infarction, unstable angina, repeat revascularization, stroke, and in-stent restenosis. We considered defining heart failure by an LVEF of 40% or less but rejected this, because left ventricular systolic dysfunction is neither necessary nor sufficient for a diagnosis of heart failure and such a surrogate would misclassify heart failure with preserved ejection fraction entirely. The incidence of heart failure is therefore not reported, and left ventricular function is described by LVEF alone.

LVEF was not part of the pre-specified covariate set and was examined post hoc in two ways. First, it was added to each fully adjusted co-primary model. Neither estimate changed materially and both remained null: acute gain β

= +0.011 mm per 1-SD (95% CI −0.073 to +0.095; p = 0.80) and late lumen loss β = −0.022 mm per 1-SD (95% CI −0.091 to +0.047; p = 0.53) (Table S3). Because LVEF was missing for 5 patients, Table S3 also reports the fully adjusted reference estimate within the identical LVEF-available subset, so that the comparison is like for like. Second, effect modification by left ventricular systolic function was examined as an interaction term and within strata defined by an LVEF of 50% (Table S4). No evidence of effect modification was found (interaction p = 0.20 for acute gain and p = 0.96 for late lumen loss), but only 15 patients (25 lesions) had an LVEF below 50% and the confidence intervals within that stratum are approximately four times wider than those of the primary analysis; that analysis is therefore uninformative rather than confirmatory and should not be read as a subgroup finding.

Left ventricular function did not differ between patients with and without QCA (62.9 ± 11.1% versus 62.7 ± 11.4%; standardized mean difference +0.02; Table S1). The selection that defines the analytic cohort, which does involve arterial stiffness, hypertension, and acute coronary syndrome, therefore does not appear to involve left ventricular function.

Table S1. Generalizability: comparison of patients with versus without QCA.

Characteristic	QCA (n = 141)	No QCA (n = 171)	SMD
baPWV, cm/s	1,637 ± 357	1,728 ± 436	−0.23
Age, years	66.0	66.5	−0.04
Male	67%	67%	−0.01
Diabetes mellitus	40%	40%	+0.01
Hypertension	66%	76%	−0.22
Current smoker	26%	27%	−0.03
eGFR, mL/min/1.73 m²	78.1	78.8	−0.03
LVEF, %	62.9 ± 11.1	62.7 ± 11.4	+0.02
LVEF < 50%	11.0%	12.0%	−0.03
Acute coronary syndrome	72%	88%	−0.41

SMD, standardized mean difference. Values are mean ± SD, mean, or %. Patients undergoing QCA had modestly lower arterial stiffness, hypertension, and acute coronary syndrome. The adjusted odds of undergoing QCA fell by 26% per 1-SD higher baPWV (odds ratio 0.74, 95% CI 0.60–0.91, p = 0.005). LVEF was available in 136 of 141 patients with QCA and 158 of 171 without QCA; LVEF percentages use those denominators. Left ventricular systolic function was near-identical in the two groups. LVEF, left ventricular ejection fraction.

Table S2. Distribution-robust analyses of late lumen loss (baPWV per 1-SD; complete cases, n = 240).

Method	β (per 1-SD baPWV)	95% CI	p	Units
Primary: cluster-robust OLS (reference)	−0.021	−0.091 to +0.050	0.57	mm
Signed-square-root transform (cluster-robust OLS)	−0.013	−0.089 to +0.062	0.73	transformed
Rank-transformed outcome (cluster-robust OLS)	−0.003	−0.041 to +0.036	0.88	rank
Huber M-estimation (cluster-robust SE)	−0.004	−0.043 to +0.036	0.86	mm

All robust specifications reproduce the null obtained in the primary model.

Table S3. Procedural, device, and left-ventricular-function covariate sensitivity analyses (baPWV per 1-SD; each covariate added to the fully adjusted model).

Fully adjusted model + covariate	Acute gain, β (95% CI; p)	Late lumen loss, β (95% CI; p)

Fully adjusted (reference)	−0.019 (−0.106 to +0.068; 0.67)	−0.021 (−0.091 to +0.050; 0.57)
+ Stent length	−0.015 (−0.096 to +0.067; 0.73)	−0.013 (−0.084 to +0.058; 0.73)
+ Stent number	−0.011 (−0.093 to +0.071; 0.79)	−0.013 (−0.085 to +0.058; 0.72)
+ Stent diameter	−0.019 (−0.100 to +0.061; 0.64)	−0.020 (−0.091 to +0.051; 0.58)
+ Lesion length	−0.027 (−0.099 to +0.046; 0.47)	−0.036 (−0.108 to +0.035; 0.32)
+ Pre-procedural diameter stenosis	−0.031 (−0.073 to +0.012; 0.16)	−0.024 (−0.094 to +0.047; 0.51)
+ DES generation (1st vs 2nd+)	−0.019 (−0.100 to +0.062; 0.65)	−0.017 (−0.083 to +0.049; 0.61)
+ Stent length, number, and diameter (block)	−0.014 (−0.096 to +0.068; 0.74)	−0.013 (−0.085 to +0.060; 0.73)
+ Left ventricular ejection fraction	+0.011 (−0.073 to +0.095; 0.80)	−0.022 (−0.091 to +0.047; 0.53)

Values are the baPWV coefficient (mm per 1-SD) after adding the listed covariate to the fully adjusted model. Acute gain was analyzed with a linear mixed model (random intercept per patient) and late lumen loss with cluster-robust ordinary least squares, the primary estimator for each outcome. The sample was 242 lesions in 141 patients, except the lesion-length rows (222 for acute gain and 220 for late lumen loss) owing to 20 missing pre-procedural lesion-length values. Every coefficient remained small and non-significant, reproducing the primary null. The left-ventricular-ejection-fraction row is based on 233 lesions in 136 patients for acute gain and 231 lesions in 136 patients for late lumen loss, because LVEF was missing for 5 patients (9 lesions); within that same subset the fully adjusted reference estimates were −0.003 (−0.087 to +0.080; p = 0.94) for acute gain and −0.031 (−0.102 to +0.039; p = 0.38) for late lumen loss, so the addition of LVEF does not shift either coefficient materially. LVEF, left ventricular ejection fraction.

Table S4. Exploratory analysis of effect modification by left ventricular ejection fraction (baPWV per 1-SD).

Analysis	Acute gain, β (95% CI; p)	Late lumen loss, β (95% CI; p)
Interaction term (baPWV × LVEF < 50%)	−0.156 (−0.392 to +0.081; 0.20)	+0.008 (−0.283 to +0.299; 0.96)
baPWV within LVEF ≥ 50%	−0.003 (−0.087 to +0.081; 0.95)	−0.015 (−0.078 to +0.048; 0.63)
baPWV within LVEF < 50%	−0.023 (−0.393 to +0.346; 0.90)	−0.109 (−0.327 to +0.109; 0.33)
Reference: fully adjusted, LVEF-available subset	−0.003 (−0.087 to +0.080; 0.94)	−0.031 (−0.102 to +0.039; 0.38)

These are exploratory, post hoc analyses; effect modification by left ventricular systolic function was not pre-specified. Models are the fully adjusted co-primary specifications (linear mixed model with a random intercept per patient for acute gain; cluster-robust ordinary least squares for late lumen loss), restricted to the 136 patients with an available LVEF (233 lesions for acute gain, 231 for late lumen loss). The interaction rows report the coefficient of the product term baPWV × LVEF < 50%. Only 15 patients (25 lesions) had an LVEF below 50%; within that stratum the confidence interval for acute gain is approximately four times wider than in the full cohort. These analyses therefore provide no evidence of effect modification by left ventricular systolic function, but they are too imprecise to exclude it and should not be read as a subgroup finding. LVEF, left ventricular ejection fraction.

Table S5. Sensitivity analyses for peripheral arterial disease and blood pressure at measurement (baPWV per 1-SD).

Analysis	Acute gain, β (95% CI; p)	Late lumen loss, β (95% CI; p)
Primary fully adjusted model (reference)	−0.019 (−0.106 to +0.068; 0.67)	−0.021 (−0.091 to +0.050; 0.57)
Excluding ankle–brachial index < 0.9 (126 patients)	−0.038 (−0.130 to +0.054; 0.41)	−0.021 (−0.086 to +0.045; 0.54)
Additionally adjusted for systolic blood pressure at measurement	−0.004 (−0.092 to +0.083; 0.92)	−0.033 (−0.118 to +0.052; 0.44)

Models are the fully adjusted co-primary specifications (linear mixed model with a random intercept per patient for acute gain; cluster-robust ordinary least squares for late lumen loss). The ankle–brachial index row excludes the 15 patients whose minimum ankle–brachial index was below 0.9 (214 lesions in 126 patients for acute gain, 212 lesions in 126 patients for late lumen loss). The blood-pressure row adds systolic blood pressure recorded at the time of the baPWV measurement to the full cohort. Both analyses reproduce the primary null.
